# How many crystal structures do you need to trust your docking results?

**DOI:** 10.1101/2025.09.19.677428

**Published:** 2025-09-24

**Authors:** Alexander Matthew Payne, Benjamin Kaminow, Hugo MacDermott-Opeskin, Iván Pulido, Jenke Scheen, Maria A Castellanos, Daren Fearon, John D. Chodera, Sukrit Singh

**Affiliations:** † Tri-Institutional Ph.D. Program in Chemical Biology, Weill Cornell Medical College, New York, New York 10065, United States; ‡ Computational and Systems Biology Program, Sloan Kettering Institute, Memorial Sloan Kettering Cancer Center, New York, N.Y. 10065, United States; ¶ Tri-Institutional Ph.D. Program in Computational Biology & Medicine, Weill Cornell Medical College, New York, New York 10065, United States; § Open Molecular Software Foundation, Davis CA, USA; ‖ Diamond Light Source, Didcot, UK

## Abstract

Structure-based drug discovery technologies generally require the prediction of putative bound poses of protein:small molecule complexes to prioritize them for synthesis. The predicted structures are used for a variety of downstream tasks such as pose-scoring functions or as a starting point for binding free energy estimation. The accuracy of downstream models depends on how well predicted poses match experimentally-validated poses. Although the ideal input to these downstream tasks would be experimental structures, the time and cost required to collect new experimental structures for synthesized compounds makes obtaining this structure for every input intractable. Thus, leveraging available structural data is required to efficiently extrapolate new designs. Using data from the open science COVID Moonshot project—where nearly every compound synthesized was crystallographically screened—we assess several popular strategies for generating docked poses in a structure-enabled discovery program using both retrospective and prospective analyses. We explore the tradeoff between the cost of obtaining crystal structures and the utility for accurately predicting poses of newly designed molecules. We find that a simple strategy using molecular similarity to identify relevant structures for template-guided docking is successful in predicting poses for the SARS-CoV-2 main viral protease. Further efficiency analysis suggests template-based docking of a scaffold series is a robust strategy even when the quantity of available structural data is limited. The resulting open source pipeline and curated datasets should prove useful for automated modeling of bound poses for downstream scoring, machine learning, and free energy calculation tasks for structure-based drug discovery programs.

## Introduction

### Structure-based drug discovery has accelerated the development of new therapeutics

**Figure 1:**
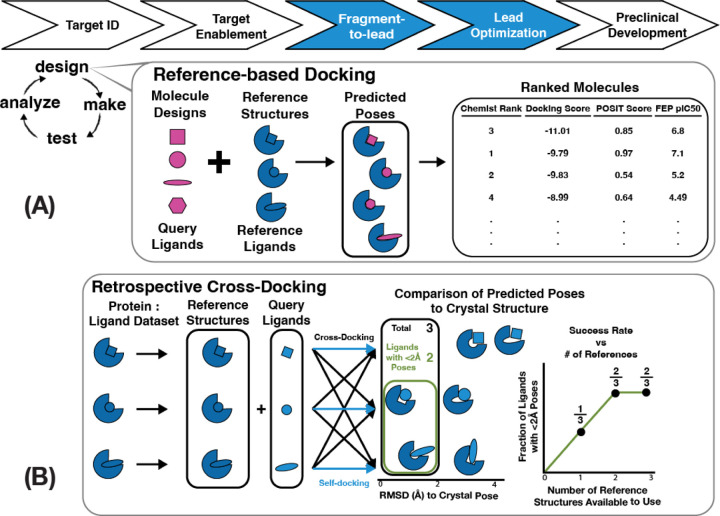
Retrospective cross-docking quantifies how pose-prediction performance depends on the number and nature of available structures. (A) Pose prediction is a critical component of the fragment-to-lead and lead-optimization stages of structure-based discovery programs (top, blue) during the design phase of the design-make-test-analyze (DMTA) cycle (left). Reference-based docking (inset) takes advantage of existing structures with reference ligands bound to the target (inset, blue) in order to pose the query ligands (inset, pink). Posed query molecules can be evaluated in multiple ways (inset, right), including structure-activity relationship rationalization by medicinal chemists, virtual screening using docking and consensus scoring functions, and binding affinity predictions using alchemical free energy calculations. (B) Retrospective cross-docking enables a thorough investigation of pose-prediction success rate. In this study, we evaluate the success rate of pose prediction as the fraction of ligands with a pose ≤ 2 Å from its experimental structure (green box). Success rate is measured for different methods for ranking the predicted poses (right).

Structure-based drug discovery (SBDD) is an established paradigm for leveraging the wealth of structural information to accelerate the development of new therapeutics.^1–4^ SBDD is founded on the idea that knowledge of the structure of a drug target—and how an early hit or lead molecule binds that target—can accelerate the rational design of potent and selective compounds on the way to preclinical development (Figure 1). The effectiveness of this acceleration is difficult to assess, as it is cost-prohibitive to run the same program in parallel with and without structural elucidation experiments. SBDD continues to become more popular since early applications to the development of HIV protease inhibitors;^2,5–7^ as of 2023, at least 65% of successful hit-to-clinical progressions used SBDD^8^ with 33% of programs from 2015–2022 using SBDD for hit-finding.^9^ As computational models improve their ability to predict binding modes and translate them into affinity or selectivity readouts, SBDD approaches can rapidly decrease time toward potency and selectivity goals in fewer iterative cycles (Figure 1).^4,5^

### The development of computational methods that use protein structures has increased the predictive power of structure-based drug design

Early SBDD efforts mainly used structures to qualitatively guide design by rationalizing the observed structure-activity relationship (SAR) of a ligand series (usually congeneric).^10^ At present, many computational methods exist that use protein-ligand structures for quantitative predictions in the broader field of computer-aided drug discovery (CADD).^11^ From empirical high-throughput virtual screening^12–14^ to physics-based simulations for binding affinity prediction^15–20^ to machine-learning models for generative chemistry,^21–24^ the toolbox of computational methods has never been larger or more diverse. Over the last five years, deep-learning methods have increasingly been applied in structure-based drug design for protein structure prediction,^25–28^ quantum mechanical functionals approximation,^29–31^ and even directing the use of these methods with active learning.^32–34^

Historically, docking, which uses a mix of physics-based and empirical scoring functions to evaluate ligand poses, has driven prediction of ligand binding modes for a congeneric series.^35–37^ This approach has become a key component of modern high-throughput virtual-screening.^14,36^ More recently, ligand-protein co-folding approaches have emerged as an alternative, though there is no standard way yet to enforce that these methods model ligands in the same binding mode as a reference structure.^35–42^ Current pose-prediction benchmarks have not comprehensively assessed the ability of co-folding methods to consistently and prospectively predict binding modes. As such, experimental structural validation continues to play a critical role in SBDD.

### Improvements in high-throughput X-ray crystallography have increased the feasibility for structure-driven drug discovery

Recent experimental advances have greatly decreased the complexity of structurally enabling a drug discovery project.^6,43,44^ Development of automated, make-on-demand chemistry, fragment-based drug discovery (FBDD), and improvements in beamline engineering and analysis have made it possible to collect hundreds to thousands of fragment-bound structures in weeks.^44–46^ Organizations such as the Diamond Light Source have pioneered these efforts, resulting in many new datasets of protein-ligand structures for therapeutically relevant targets.^47–53^

Despite substantial advances, crystallography remains a costly and time-consuming endeavor with potential bottlenecks throughout the process. Producing a protein sample that is both biochemically relevant and crystallizable is often a major hurdle for each new project. Even once obtained, identifying crystallization conditions that yield suitable crystals introduces another layer of unpredictability. Added to this are the logistical challenges of securing regular beamtime and shipping samples to synchotron facilities. These barriers are well recognized in the SBDD community and raise the central questions we address this in this work:^54–56^ *How many structures are needed to drive progress with computational models? Can we define a point of diminishing returns, or is more structural data always better?*

### Retrospective cross-docking enables evaluation of reference-based pose prediction

As accurate pose prediction is foundational to SBDD efforts, we evaluate the gained utility of additional crystal structures by examining how available structures affect pose prediction.^57^ We distinguish pose-prediction as its own task from virtual screening, which typically refers to selecting a subset of molecules from a database of candidates for testing in a downstream assay, whereas pose-prediction refers to an *in-silico* determination of the 3D coordinates of a protein-ligand complex for a selected ligand. Ligand-based virtual screening assesses whether proposed candidates are likely to bind to the target using only molecular properties. Structure-based virtual-screening takes advantage of SBDD by both predicting the pose of the ligand in the target and using a scoring function to rank the posed molecules. Pose-prediction was first developed as the DOCK software in 1982,^58^ and several of these early docking algorithms are still in use today.^59–61^ Algorithmic^62^ and scoring^63,64^ improvements have increased the speed, versatility, and accuracy of these programs. One of the early examples of ‘reference-based’ docking, shown by OpenEye’s HYBRID method, improves traditional (protein-only) docking algorithms (OpenEye FRED) by combining structure-based and ligand-based scoring functions (hybrid docking). HYBRID docking uses a modified scoring algorithm that prioritizes query ligand poses which maximize the overlap with the crystallographic ligand. ^65,66^ OpenEye’s POSIT algorithm improves upon hybrid docking by evaluating poses using the POSIT Probability, an estimate of the likelihood that the ligand will bind within 2 Å of the predicted pose.^67^ OpenEye’s pose prediction methods are readily available for academic testing and benchmarking, providing an industry-standard suite to assess pose-prediction.

Reference-based (also called templated, or template-based) docking is primarily useful during the hit-to-lead (or fragment-to-lead, in FBDD) and lead optimization phases of the drug discovery pipeline when crystal structures are available (Figure 1A). Pose prediction models are usually evaluated in self-docking experiments, where ligands from protein-ligand crystal structures are redocked into their corresponding crystal structures structures.^66,68^ Performance is evaluated by computing the heavy-atom root-mean-squared deviation (RMSD) of the predicted poses to their crystallographic poses. ^66,68^ Traditional pose prediction has considered an RMSD ≤ 2 Å as a sufficiently accurate binding pose, in part due to the resolution ranges found in many ligand-bound crystals.^61^ Although self-docking studies benefit from datasets with only a single structure per ligand, they cannot evaluate cases in which structures of related ligands are available. Cross-docking on the other hand, mimics real-world scenarios by docking each ligand from a dataset of protein-ligand structures to every other protein structure, enabling a comprehensive analysis of pose prediction performance (Figure 1B). Few publicly available datasets that reflect a real hit-to-lead optimization pipeline (”lead-opt” datasets) are rich in both binding affinities and crystal structures. As an exception, kinase domains have been abundant in public datasets as they readily crystallize and are a thoroughly-mined target for drug discovery given their prevalence in many therapeutic areas.^69,70^ The Volkamer Lab recently ran a retrospective cross-docking analysis of over 423 kinase inhibitors bound to 10 different kinases.^71^ They found that POSIT outperforms FRED for similar reference and query molecules, and that there are diminishing returns in performance gain after 20–50 structures.^71^ We expand on this work by drawing upon the largest set of publicly available protein-ligand structures for a single target, over 400 crystal structures, generated by the COVID Moonshot Consortium.^46,72^

### The COVID Moonshot provides an unprecedentedly large dataset for cross-docking evaluation

**Figure 2:**
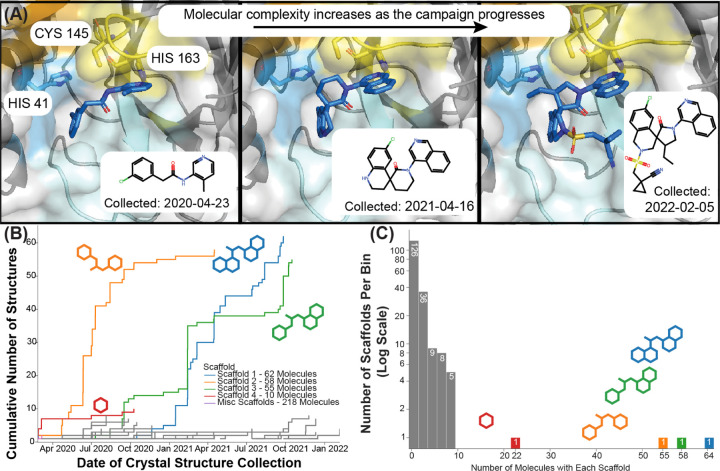
The COVID Moonshot SARS-CoV-2 main protease protein-ligand dataset shows a real-world scenario where the ligands become more elaborated over time. (A) The SARS-CoV-2 main protease binding site in three example crystal structures from the COVID Moonshot showing how the designed ligands increase in degree of elaboration over time. The catalytic dyad of His41 and Cys145, as well as a key P1 residue His163 are shown (sticks). The protein surface is colored by the protease subpocket names S1’ (orange), S1 (yellow), S2 (blue), and S3–5 (cyan). (B) Empirical cumulative distribution of the number of structures per generic Bemis-Murcko scaffolds (corresponding color) over time during the COVID Moonshot campaign. (C) Histogram of the number of molecules per Bemis-Murcko scaffold. Four scaffolds (colored as in B) account for the majority of the dataset, with the remaining scaffolds (gray) representing less than ten molecules each. A significant number of molecules (126) are singlets, not sharing a generic Bemis-Murcko scaffold with any other molecule in the dataset.

The COVID Moonshot Consortium, a global citizen science effort to combat the SARS-CoV-2 pandemic, started when the Diamond Light Source deposited a crystallographic screen of 71 fragments to the SARS-CoV-2 Main Protease (Mpro) in March 2020.^73^ With the goal of developing these early hits into a potential drug candidate, the Consortium assayed potential hits sourced from worldwide contributors, resulting in potent and developable molecules.^74^ With increased funding, personnel, and scope, the ASAP Discovery AViDD center progressed this program into preclinical studies,^75^ in addition to developing several other discovery programs.^51,52,76^ In doing so, the AViDD center generated an unprecedentedly large, publicly available dataset of protein-ligand crystal structures. This dataset is unique not only for its size, but it also captures a nearly complete drug discovery program from early fragments to optimized leads including progressively more elaborated chemical series developed over time (Figure 2).

The COVID Moonshot dataset represents a promising model system for evaluating reference-based docking performance due to the favorable biophysical properties of the SARS-CoV-2 main protease as an SBDD target.^72^ The SARS-CoV-2 main protease has been the subject of years of inhibitor development studies, with nearly 20 drugs in the market or clinical trials.^75,77,78^ It is a protease with a large, conserved set of protein cleavage sequences, presenting a druggable binding site with many features that have been used to optimize affinity and selectivity.^79^ Relative to other well-studied inhibitor targets, Mpro is readily crystallizable without dramatic modification, amenable to crystal soaking, and the binding site does not undergo any significant conformational changes upon inhibitor binding across different chemical series.^73,80^

In this work, we use 403 protein-ligand structures from the COVID Moonshot to quantify how the success rate of accurately predicting ligand poses depends upon the quantity of available structural data. We show that only a small fraction of available structures per ligand series is needed to achieve near-maximal performance; we note that pose prediction success continues improves with additional structures. We examine how success rate, defined as pose-prediction within 2 Å of the known pose, varies when only using structures in the order they were collected, replicating a realistic drug discovery campaign. Finally, we explore the consequences of ligand diversity in the structural dataset, identifying how diminishing returns are most evident when collecting additional structures of a particular scaffold. In doing so, we assess which structures are most valuable and identify potential strategies for most effectively leveraging available structural data to support decision making. In turn, we use the data of a real-world open-science drug discovery campaign to assess when collecting additional structural data for a particular ligand series offers diminishing returns.

## Results and Discussion

### Reference-based docking outperforms standard docking, with the biggest improvement found for ligands docked to similar references

The dataset of crystal structure from the COVID Moonshot was downloaded via Fragalysis on April 1st, 2024.^48^ These 803 crystal structures were filtered to exclude duplicates of the same ligand and to include only non-covalent ligands from the Moonshot discovery project which bind to the active site. This resulted in a dataset of 414 crystal structures. Of these, 403 of were successfully prepped and cross-docked using the OpenEye toolkits as implemented in the *drugforge* software package (see Detailed Methods). We refer to the ligand to be posed as the **query ligand**, the crystal structure used in docking as the **reference structure**, and the crystal structure ligand as the **reference ligand**. We used the POSIT algorithm to generate poses of the 403 query ligands against each of the 403 reference structures using either the FRED-only or full-POSIT settings, creating two datasets of containing 162,006 query-reference ligand pairs with 1 pose each.

**Figure 3:**
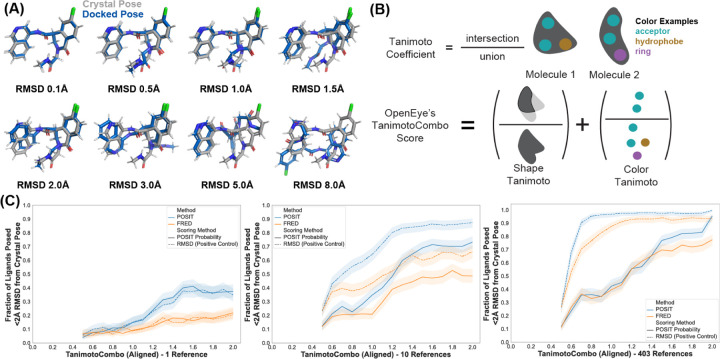
Pose prediction accuracy improves with increasing ligand similarity to the reference structure. (A) Examples of docked ligand poses (blue) with increasing RMSD to the crystallographic pose (grey) for an example ligand (MAT-POS-a54ce14d-2). (B) The TanimotoCombo Score used by OpenEye’s POSIT docking algorithm combines the Shape and Color Tanimoto Coefficients to provide a scaffold-independent, spatially-aware measure of chemical similarity (see Methods). (C) Pose prediction success is plotted as a function of the similarity of the query ligand to the reference ligand, comparing two docking methods: POSIT (blue) and FRED (orange), and two scoring methods: the RMSD as a positive control (dashed line) and the POSIT Probability (solid line). The success rate is shown for three examples, in which 1, 10, or all available structures (403) are available to use. For each query ligand, the structures are first filtered by similarity, and then the number of references indicated are randomly sampled from the remaining structures by bootstrapping 1000 times. Although POSIT outperforms FRED for similar molecules, this relative improvement decreases when more structures are used.

For each query ligand, we rank the resulting poses by either the POSIT Probability or by the true RMSD (a positive control) and return the top-ranked pose to evaluate pose prediction success. We define the success rate for pose-prediction as the fraction of query molecules whose top-ranked pose has ≤ 2 Å heavy-atom RMSD to its crystallographic pose, a typical performance metric for pose-prediction studies (Figure 3A).^61,67,70^ RMSD provides a practical performance metric for our ∼300,000 ligand poses: RMSD is computationally efficient, scales with ligand size, and enables comparison across diverse molecular scaffolds. We note that RMSD as a performance metric is limited as it does not differentiate thermodynamically favorable poses from unfavorable one nor does it consider chemical interactions that favor binding. In other words, a 0.1 Å RMSD pose might contain an implausibly high-energy clash, and a 3 Å RMSD pose might retain all important interactions. RMSD is also blind to the difference between the motions of a flexible substituent exposed to solvent (which may be well-tolerated) or in a constrained pocket. Finally, similar conformational differences can have different RMSD values depending on the total ligand size. For example, given equal motions of a benzene ring in a small fragment and a large ligand, the RMSD of the small fragment will be larger. As such, while RMSD is an imperfect metric that requires careful consideration during interpretation, it remains a tractable, scalable, and efficient metric for our diverse library of chemical moieties.

We measure the success rate as a function of the aligned TanimotoCombo similarity for both the FRED and POSIT docked poses (Figure 3C) and find that the performance of reference-based docking (POSIT) outperforms standard docking (FRED) as the molecules become more similar.^71^ We first reproduce previous findings that show that POSIT outperforms other docking methods when docking to similar reference ligands (Figure 3C).^67,71^ While FRED relies solely on protein-ligand interactions, POSIT incorporates Tanimoto-Combo scoring to align the query ligand with the reference (crystal structure) ligand. We would expect the POSIT-posed success rate to increase more than the FRED-posed success rate as a function of increasing query-to-reference ligand similarity since the protein structures in this dataset are quite similar to each other. This expectation holds when using a single reference structure (Figure 3C, left panel), where the improvement in the success rate as a function of the ligand similarity is larger for full POSIT (blue, 0.05 to 0.35) than for FRED (orange, 0.05 to 0.15). However, when all possible references are included, the performance is equivalent until the similarity is *>*0.8 (Figure 3C, right panel). This suggests that FRED’s performance approaches that of POSIT when FRED is given enough opportunities to generate poses via having additional reference structures.

We examine a few ways of measuring chemical similarity, including the TanimotoCombo score from OpenEye (Figure 3B),^67^ the maximum common-substructure (MCS) (SI Figure 1A),^81^ and the extended-connectivity fingerprint (ECFP)^82^ (SI Figure 1B). The aligned TanimotoCombo and MCS Tanimoto coefficients (Figure 3B, Methods) describe the dataset as being less diverse, while the unaligned TanimotoCombo, ECFP4 fingerprint, and ECFP10 fingerprint describe the dataset as being more diverse (SI Figure 1C). The ECFP Tanimoto measures chemical similarity more in terms of the presence of functional groups and local motifs, while the MCS Tanimoto is more sensitive to changes in the core scaffold. Since a majority of core scaffold diversity stems from variations of the decorating groups, the fingerprints report greater ligand diversity than MCS Tanimoto. We use the TanimotoCombo as our measurement of chemical similarity, as it provides the widest range of ligand similarity scores, avoids the idiosyncratic behavior and feature choices of the MCS, and takes advantage of available 3-dimensional information.

The discrepancy between the POSIT Probability (Figure 3C, solid lines) and the true RMSD (Figure 3C, dashed lines) increases when more references are used. When 10 references are available, the maximum difference between the POSIT Probability (0.25) and the true RMSD (0.6) is at a TanimotoCombo similarity of 0.8, with a difference of 0.35. This difference grows to 0.6 when all 403 reference structures are used. This suggests an improved scoring method for for query-reference pairs with a TanimotoCombo of *<*0.8 would be the most impactful. We also performed this comparison with the Tanimoto of the ECFP4 2048 bit fingerprint as implemented in RDKit (SI Figure 1D). Upon analyzing the success rate as before, we observe that the success rate plateaus after similarities of ∼0.4, consistent with prior results (SI Figure 1C). There are few query-reference pairs with an ECFP4 similarity of *>*0.4. Thus, we suggest against using ECFP4 as a similarity metric for analyzing single-target datasets, as it conflates the chemical diversity represented by the dataset.

It is worth noting that success rate includes structures drawn from the whole dataset, which does not reflect the prospective nature of a drug discovery campaign. Rather, the dataset of a successful drug discovery campaign will inherently converge into a smaller subset of molecule designs. As such, prospective assessments must consider the time-evolution of ligand design. To appropriately estimate the prospective performance of reference-based docking, we consider the time-evolution of the Moonshot dataset by comparing a random selection of the structures to a selection ordered by the date of crystal structure collection.

### Pose prediction success has diminishing returns with an increasing number of available crystal structures

**Figure 4:**
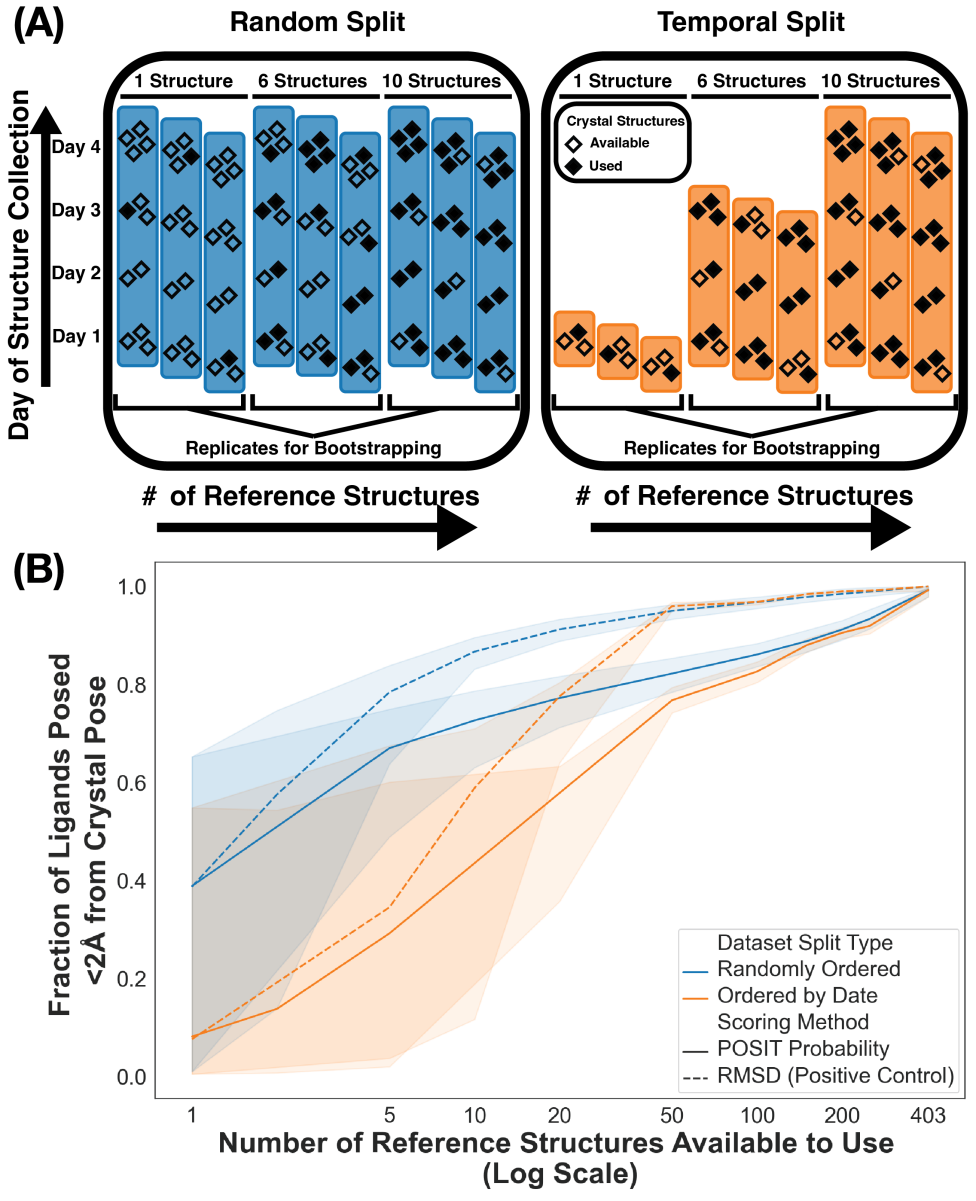
Pose prediction is more challenging when limited to the first crystal structures collected during the campaign. (A) Schematic representation of the two dataset splits. For the Random Split (blue, left), reference structures are randomly selected from the entire dataset (highlighted blue regions), whereas for the Temporal Split (orange, right), possible reference structures are selected chronologically by increasing the last date of crystal structure deposition, with same-day structures randomized. For both splits, the confidence interval is reported by bootstrapping over 1000 samples. (B) The success rate (Fraction of Ligands Posed ≤ 2 Å from the Crystal Pose) for both dataset splits are reported as a function of the number of reference structures available for pose prediction. Success rate is shown for both the Random Split (blue) and Temporal Split (orange) and for poses ranked either by the RMSD to the crystal pose (solid line) and the POSIT Probability (dotted line).

To assess how increasing the number of crystal structures improves pose prediction performance, we compare two splits: Structures were ordered either randomly (Random Split) or chronologically by collection date (Temporal Split) then incrementally added to the pool of available structures for each reported success rate (Figure 4A). Performance is better for the randomly shuffled dataset (Figure 4B) since randomly shuffling structures increases the probability of the query and reference molecules having a high similarity.

With the first 50 crystal structures collected during the COVID Moonshot Initiative (in the Temporal split), 75% of the ligands can be posed within 2 Å of their crystallographic pose using the POSIT docking algorithm (Figure 4B, orange solid line). As before, we compare ranking the predicted poses for each ligand by the POSIT probability to ranking them by the RMSD as a positive control. For the RMSD-ranked poses, the difference in success rate between the two structure-splits is *<*5% after ∼20 structures, but it takes ∼120 structures for the POSIT-ranked poses to match the performance of the RMSD-ranked poses.

There is a large distribution of success rates for the Temporal Split when using *<*10 structures (Figure 4B, orange lines). Some initial structures result in success rate *<*10% independent of ranking by RMSD or POSIT probability, suggesting that initial failures are due to posing rather than scoring.

Although only an eighth of the dataset is needed to get above a 70% success rate in the Temporal Split, some ligands are not posed correctly until the entire dataset of structures is used. To further examine the causes of these failed pose-prediction tasks, we first explored how success rates depend on the scaffold used.

### Few structures per scaffold are needed to correctly pose a candidate molecule

**Figure 5:**
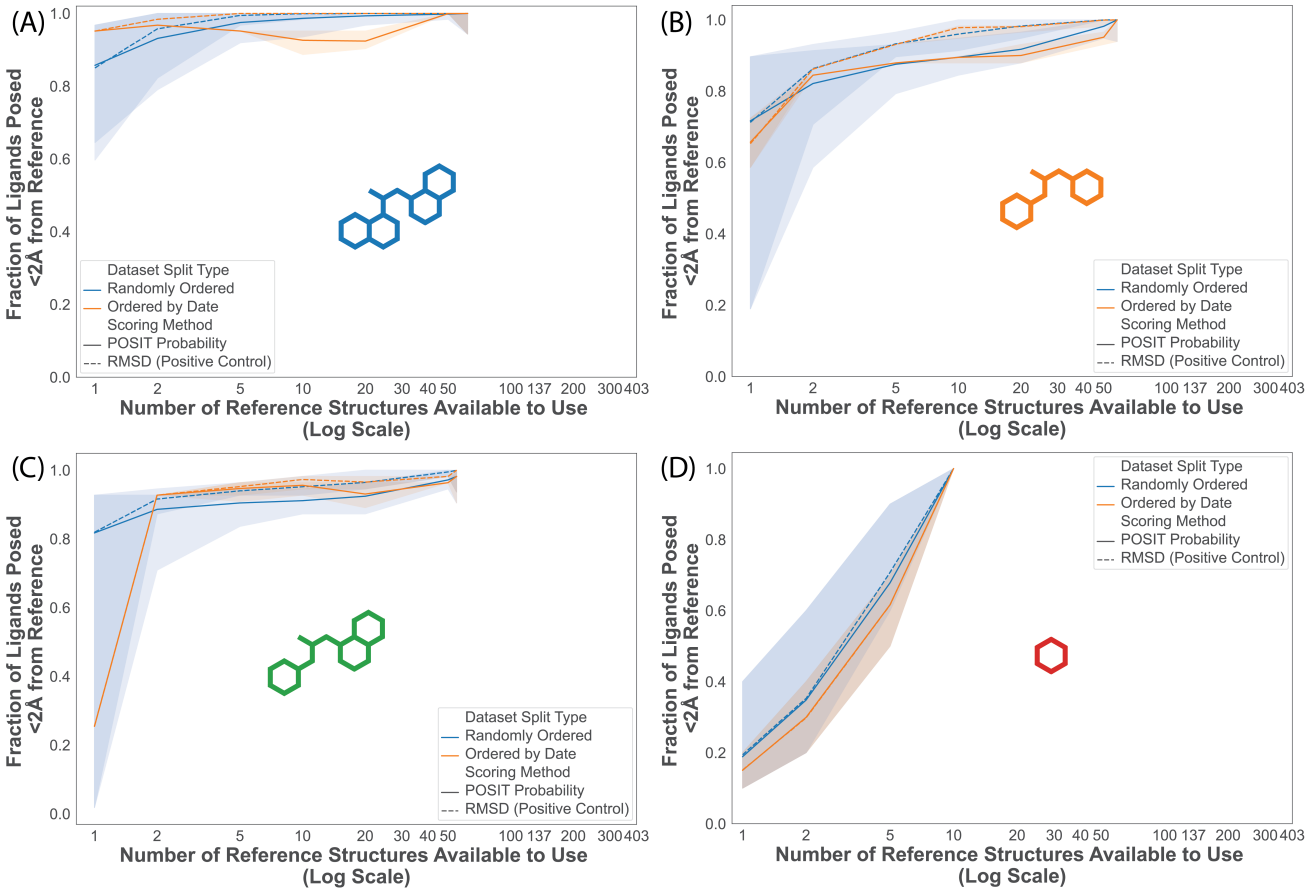
Pose prediction success is higher when the reference and query have the same scaffold, unless the scaffold is a single-ring fragment. The success rate (Fraction of Ligands Posed ≤ 2 Å from the Crystal Pose) of scaffolds 1–4 (A-D, respectively) are reported as a function of the number of reference structures available for pose prediction. The total number of structures is different for each scaffold but they are plotted on the same scale as Figure 4 for clarity. Success rate is shown for both the Random Split (blue) and Temporal Split (orange) and for poses ranked either by the RMSD to the crystal pose (solid line) and the POSIT Probability (dotted line).

We hypothesize that the main source of diminishing returns stems from adding structures with the same generic Bemis-Murcko scaffold. That is, for an individual scaffold, additional structures collected do not significantly improve success rate and rather offer costlier diminishing returns. For robust statistics and representation, we limit this analysis to scaffolds with at least 10 molecules represented in this dataset, which include the top four most common scaffolds (Figure 2C). We calculated the success rates for these four scaffolds docked only to structures with the same scaffold. Even with only 1 structure, the success rates were higher for the first three scaffolds than for the full split (Figure 5A–C). For these three scaffolds, we observe minimal improvement in success rate after the second structure is added suggesting that additional corresponding structures of a given scaffold are not needed to achieve a maximal success rate. On the other hand, scaffold 4, constituting single ring fragments, is an outlier. Each added structure provides a significant improvement in the fraction of ligands posed within 2 Å of the crystal pose (Figure 5 D). This is consistent with previously observed challenges in fragment pose prediction tasks.^83–85^

**Figure 6:**
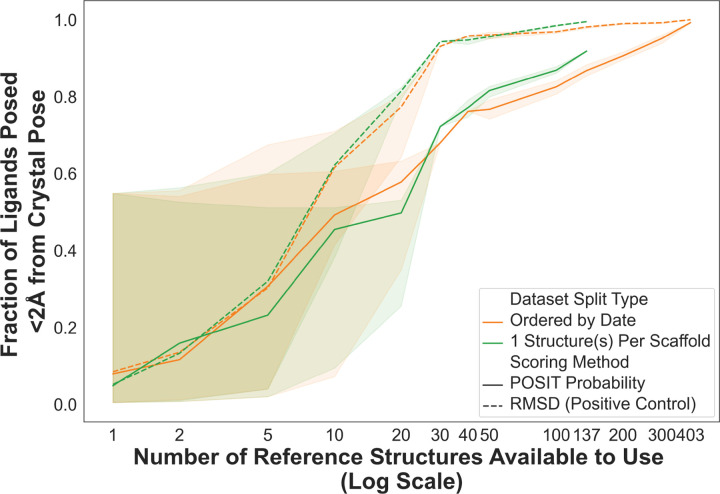
Collecting only 1 structure per scaffold shows a minor efficiency improvement from the temporal split beyond 50 structures. Pose success rate (fraction of ligands posed ≤ 2 Å from the crystal pose) is reported as a function of the number of reference structures available for pose prediction. Success rate is shown for the previous Temporal Split (orange) and when a single structure per scaffold is made available (green) for poses ranked either by the RMSD to the crystal pose (solid line) and the POSIT Probability (dotted line). The structures for the One Structure Per Scaffold Split are made available in order of their collection date, as in the Temporal Split.

We assess whether pose prediction performance can be maximized in sparse data regimes by limiting the number of structures collected for any ligand scaffold. We analyze this strategy (Figure 6, green lines) in comparison to the Temporal Split (Figure 6, orange lines). Below 50 structures, the efficiency between the different strategies remains similar, while we do see a small improvement in efficiency beyond 50 structures. However, the success rate has already reached ∼80% when over 50 structures are present leaving only minimal room for improvement. To examine whether additional structures per scaffold improve the performance, we simulate collecting an increasing number of structures per scaffold, from 1 to 20, evaluating efficiency as a function of the number of available structures. We do not see any significant improvement in efficiency as the number of structures per scaffold increases (SI Figure 7).

To further probe whether any particular scaffolds were more challenging than others, we analyze each crosswise query-reference pair for the top 20 scaffolds, ranked either by RMSD (SI Figure 4A) or by POSIT Probability (SI Figure 4B). By averaging the results we see that for all the scaffolds, predicting their poses was more successful than using their crystal structures as the reference (SI Figure 5).

As observed previously, the single ring fragment (scaffold 4) remains challenging both as query and as reference (SI Figure 7, top left). Scaffold 6 (SI Figure 7, top row, middle) was also challenging for both, likely due to the extended linker between the central amide bond and the single ring system, giving it increased flexibility. Scaffold 8 (SI Figure 7, top right) is unusual in this dataset as it contains a *β*-lactam ring, which may have contributed to its challenges for pose prediction. Scaffold 14 (SI Figure 7, bottom left) is also notable as it contains one of the few examples of a branching point with a tertiary carbon. This moiety was also explored with scaffold 33 (SI Figure 7, bottom right), which also performed poorly.

In conclusion, we identify a point of diminishing returns by limiting our dataset to 1–2 structures per generic Bemis-Murcko scaffold. However, for a project such as the COVID Moonshot which generated *>*100 generic Bemis-Murcko scaffolds, this would still require collecting *>*100 structures. To avoid having to collect *>*100 structures, we wanted to find a set of 5–10 structures with a high success rate for the whole dataset. We previously showed that only a few structures per scaffold are needed to pose the 3 most common scaffolds with *>*95% success rate (Figure 5A–C). Therefore, we next evaluated how ligands from the remaining scaffolds perform when docked to these structures (SI Figure 6). With a success rate of 60% at 10 structures, collecting several structures of the top three scaffolds is a reasonable strategy for using a minimal number of structures to achieve the best possible performance. However, it remains unclear whether docking failures are primarily due to failures in pose sampling, or in failures to rank multiple poses.

### Keeping up to 50 poses from POSIT shows a minimal improvement in success rate even with a perfect ranking

**Figure 7:**
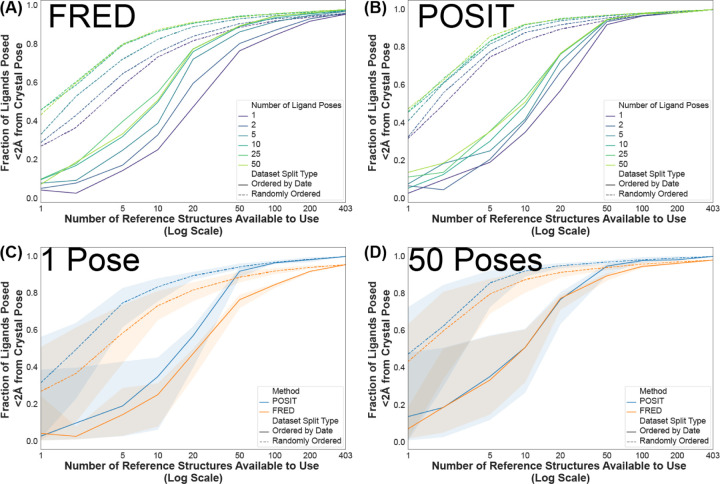
The lowest RMSD pose generated by the POSIT algorithm may not be ranked best by the POSIT probability. (A) and (B) The success rate (fraction of ligands posed ≤ 2 Å from crystal pose) is reported as a function of the number of reference structures available to generate up to 50 ligand poses using either FRED (A) or POSIT (B) and ranked by RMSD. The number of poses generated range from 1 (purple) to 50 (lime green) and structures used were using either a Random Split(dashed line) or a Temporal Split (solid line), as in Figure 4. (C) and (D) The same data is plotted for 1 pose (C) and 50 poses (D) for FRED (orange) and POSIT (blue) generated poses for the random (dashed line) or ordered-by-date (solid line) structures. These results show that ranking up to 50 poses by even a perfect scoring function (RMSD, the same function we use to measure success) only increases the success rate by 10%, and with more than 50 structures there is no significant improvement to generating more poses. The improvement in the success rate is more significant for the FRED-posed structures (20% → 50%) than the POSIT-posed structures (30% → 50%).

So far we have only analyzed pose prediction results where a single pose is returned by the docking algorithm. Retrieving multiple poses for each protein-ligand complex hurts scalability and risks a computational pipeline becoming prohibitively expensive for a real-world drug discovery campaign. To test whether the failures stem from poor initial pose generation or inaccurate POSIT probability scoring, we had POSIT return up to 50 deduplicated poses for each query–reference combination (see Detailed Methods). The success rate is plotted, as before, as a function of the number of reference structures available to use for either the Random or Date Split (Figure 7). Since the POSIT Probability is used to rank poses by default, we report the positive control (RMSD-ranked) success rate. As before, the Random Split has a higher success rate than the Date Split, and reference-based docking (POSIT) outperforms protein-only docking (FRED). Increasing the number of poses returned by the docking algorithm from 1 to 50 improves the success rate, with the greatest improvement for both methods occurring when 5–20 structures are made available. FRED shows a 20% improvement and POSIT has a 10% improvement in pose prediction success rate. This effect is smaller than that seen for adding more structures, matching results shown in previous kinase cross-docking benchmarks. ^71^ Our results highlight the importance of pose-sampling; with current pose sampling strategies, a perfectly predictive scoring function would only improve success rate by 10–20%. Currently, POSIT fails to sample any correct poses for at least 60% of ligands when only docking to the first 5–20 crystal structures in the Temporal Split.

## Conclusion

In this work, we use a publicly visible drug-discovery-campaign—the COVID moonshot—to retrospectively evaluate the effect of the number of available crystal structures on pose prediction performance. We find that only a few structures of any scaffold-bound target are needed to successfully predict the poses of future candidate molecules later in the campaign. This performance is dependent on the similarity of the reference to query ligand. While reference-based docking (POSIT) significantly outperforms protein-only docking (FRED) when only a few structures are available, the difference becomes less significant as more structures are included. Although the fraction of correctly predicted poses increases with the number of structures used, this improvement results in diminishing returns. Only a fraction of available structures are needed to approach maximal performance. Predicting the pose of a molecule with a given generic Bemis-Murcko scaffold is most successful when docking to a structure with that same scaffold. Including additional structures with the same scaffold does not typically enhance performance. The POSIT Probability is moderately predictive of pose prediction success, with failures stemming from poor pose generation (Figure 4B). In the Date Split (Temporally Ordered), the RMSD-ranked success rate is only ever about 20% better than the POSIT-Probability ranked success rate. Our pipeline-driven analysis highlights that while few structures are needed to pose 70% of ligands accurately, a poor match between the reference ligand and the query ligand results in the majority of failure modes. It is worth noting that the success rate required will likely depend on the specific target and the downstream computational method being used.

In conjunction with our retrospective analysis, we provided the baseline pose-prediction model for a prospective blind-challenge for SARS-COV-2 and MERS-CoV pose prediction, in collaboration between Polaris, OpenADMET, and ASAP Discovery.^86^ Although our docking workflow was the best performing non-co-folding model, the top three best performing models used co-folding.^86^ Similarly, co-folding models have recently been shown to be competitive with docking models for preparing structures for free energy calculations although at much greater computational cost.^87^

Future extensions of our work would evaluate the impact of pose prediction performance on the accuracy of alchemical free energy estimation.^57^ Additionally, future work will explore the use of protein-ligand interaction fingerprints to evaluate pose prediction performance.^88,89^

Future versions of our analysis would benefit from large datasets of protein-ligand structures for more challenging targets. While large amounts of structure datasets are more accessible to academic groups than been before, many proprietary protein-ligand structural datasets are maintained by pharmaceutical companies.^90^ Thus, a vast majority of single-target protein-ligand datasets, and the downstream discoveries they enable, are locked away as closed-source repositories. However, there is ample evidence that availability of protein-ligand structural datasets enables discovery; compounds from the COVID Moonshot were the foundation for other groups to develop their own SARS-CoV-2 Mpro inhibitors.^91^ Realizing this, several of these companies have joined together to use ”federated” machine learning on their private datasets, enabling them to benefit from this vast source of data without needing to disclose it.^92,93^ Academic groups such as the Structural Genomics Consortium^94–96^ and the newly minted OpenBind Consortium^47,97^ are working towards generating large datasets of protein-ligand structures for clinical targets in an open-source, open-science manner. We hope this contribution to the field will help guide these groups to collect the structures which provide the most value for structure-based drug discovery.

## Detailed Methods

### SARS-CoV-2 Protein-Ligand COVID Moonshot Dataset

The dataset of 803 protein-ligand crystal structures collected by the COVID Moonshot Consortium were downloaded from the Fragalysis website using their API on 2024/04/01 using scripts from the *asapdiscovery* software repository. Of these, just the 366 X-series and 265 P-series structures were further filtered by selecting only the *_0A structures, which contain all crystal structures in which the ligand is bound to Chain A of the SARS-COV-2 Main Protease. To further refine this, only the structures in which the ligand is bound to the active site of the protein were selected, yielding 543 protein-ligand crystal structures. Structure Preparation with Spruce TK The structures were prepared using the OpenEye Toolkit (version 2022.1.1) according to the following steps: 1) building in sidechains, loops, missing residues, and hydrogens, and 3) protonating any protonatable residues. This preparation was performed using code from the asapdiscovery toolkit.

### OpenEye Docking

OpenEye’s POSIT algorithm was used to dock the structures, with helper code from the asapdiscovery toolkit. This is a rigid-body docking algorithm that uses either FRED, HYBRID, or SHAPEFIT algorithms from OpenEye depending on how similar the query ligand is to the reference ligand. Although multiple structures can be passed to POSIT to enable automated choice of optimal reference structure, for the cross-docking analysis, each pairwise combination of ligand and protein were docked separately, resulting in 162,409 (403^2^) potential docked structures. The ligands were passed to this docking function as 2D SDF files to avoid biasing the docking results with the crystalized conformation of the ligand. For the multiple-pose analysis, 50 poses for each protein-ligand combination were requested from POSIT, however in many cases, the algorithm will return less than the requested number of poses, with some being duplicates. To account for this, all poses were ranked according to POSIT probability, and any pose with an RMSD ≤ 2 Å to any other, higher ranking pose was removed, resulting in a set of deduplicated poses. This is motivated by the fact that since RMSD follows the triangle inequality, if pose A is ≤ 2 Å to the crystal structure pose, and ≤ 2 Å to pose B, then pose B is also ≤ 2 Å to the crystal structure.

### Chemical Similarity Analysis

#### Maximum Common Substructure (MCS)

The Maximum Common Substructure was calculated for all ligand pairs using the OpenEye python toolkit using default settings. The MCS Tanimoto Coefficient was calculated as the number of atoms in the MCS divided by the total number of atoms in both ligands minus the number of atoms in the MCS.


(1) $$\text{MCS}\;\;\text{Tanimoto}=\frac{\text{N}\;\;\text{Atoms}\;\;\text{in}\;\;\text{MCS}}{\left(\text{Total}\;\;\text{Atoms}\;\;\text{in}\;\;\text{A}\right)+\left(\text{Total}\;\;\text{Atoms}\;\;\text{in}\;\;\text{B}\right)-\left(\text{N}\;\;\text{Atoms}\;\;\text{in}\;\;\text{MCS}\right)}$$

#### Extended-Connectivity Fingerprint (ECFP)

The ECFP was calculated for all ligand pairs using the OpenEye python toolkit. The results shown in this paper were calculated with a radius of 2 (ECFP4) or 5 (ECFP10) and a bit size of 2048. The Tanimoto Coefficient was calculated with the OpenEye function OETanimoto.

#### TanimotoCombo Score

The TanimotoCombo score was calculated for all ligand pairs using the OpenEye python toolkit. The TanimotoCombo Score used by OpenEye’s POSIT docking algorithm combines the Shape and Color Tanimoto Coefficients, which provides a scaffold-independent, spatially-aware measure of chemical similarity. The shape is defined by the combined Gaussians used to represent the ligand atoms, and the color is defined by the Mills Dean Implicit Force Field and includes terms such as hydrogen bond donor, acceptor, rings, cation, anion, and structural. Two variations of this calculation were performed. The TanimotoCombo (Aligned) attempts to minimize the TanimotoCombo between the two ligands by altering the position, but not conformation of the molecules. The unaligned version takes the atomic coordinates as given. Here we use the former as it represents what we use prospectively.

### Bemis-Murcko Scaffold Clustering

The generic scaffold as implemented by RDKit was generated for all ligands from the Moonshot dataset, and all ligands sharing the same scaffold are clustered together.

### Scoring

Docking results were scored using OpenEye’s POSIT and Chemgauss4 scores, as well as the RMSD of the docked structure to the known structure.

#### POSIT Score

The OpenEye POSIT score is the estimated probability that the pose of the molecule is ≤ 2 Å RMSD to the known pose, given that the ligand binds the target. It is not intended to predict binding affinity as a comparison between different ligands, but rather identify the best pose for the ligand.

#### Chemgauss4 Score

Chemgauss3 uses Gaussian smoothed potentials of the following interactions: shape, hydrogen bonding between ligand and protein, hydrogen bonding with implicit solvent (i.e. in aqueous phase), and metal-chelator interactions. The Chemgauss4 score is a modification of the Chemgauss3 scoring function with improved hydrogen bonding terms. The Chemgauss4 score is meant to correlate with binding affinity (although, like most docking scores, usually does not).

#### RMSD Calculation

All RMSDs presented herein were calculated using the OpenEye toolkit’s *OERMSD* function on the ligand heavy (non-hydrogen) atoms, which takes into account automorphisms—symmetrical permutations of the ligand configuration which, if not taken into account, resulting in incorrectly high RMSD for chemically equivalent poses.^98^

### Success Rate and Bootstrapping

The Success Rate, as used in this paper, is calculated by calculating the fraction of query ligands posed ≤ 2 Å to their original crystal pose. When possible, the confidence interval for this value is reported by performing this calculation 1000 times, using randomized references and then taking the values at the 2.5% and 97.5% percentiles as the lower and upper CI values. Where there is not an easy way to randomize the references, we use the beta function to get the posterior probability of observing the number of successes and failures.

## Supplementary Material

Supplement 1

## Figures and Tables

**Figure 8:**
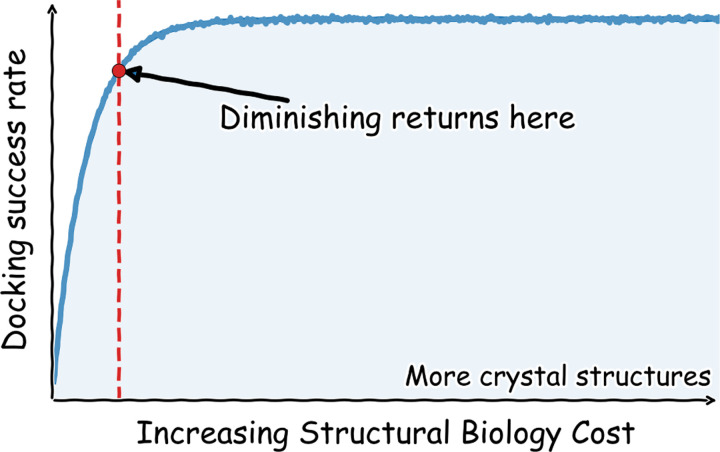
Table of Contents graphic
