# Supplementary material for: How many crystal structures do you need to trust your docking results?
Source: bioRxiv. 2025 Sep 24:2025.09.19.677428. Preprint. [Version 1] doi: 10.1101/2025.09.19.677428 (PMC12485767; doi:10.1101/2025.09.19.677428)
Supplement: Supplement 1 [file media-1.pdf]

Supporting Information: How many crystal structures do you need to trust your docking results?

Alexander Matthew Payne,[†,‡] Benjamin Kaminow,[¶,‡] Hugo MacDermott-Opeskin,[§] Iván Pulido,[‡] Jenke Scheen,[§] Maria A Castellanos,[‡] Daren Fearon,[‖] John D. Chodera,[*,‡] and Sukrit Singh[*,‡]

†*Tri-Institutional Ph.D. Program in Chemical Biology, Weill Cornell Medical College, New York, New York 10065, United States*

‡*Computational and Systems Biology Program, Sloan Kettering Institute, Memorial Sloan Kettering Cancer Center, New York, N.Y. 10065, United States*

¶*Tri-Institutional Ph.D. Program in Computational Biology & Medicine, Weill Cornell Medical College, New York, New York 10065, United States*

§*Open Molecular Software Foundation, Davis CA, USA*

‖*Diamond Light Source, Didcot, UK*

E-mail: john.chodera@choderalab.org; sukrit.singh@choderalab.org

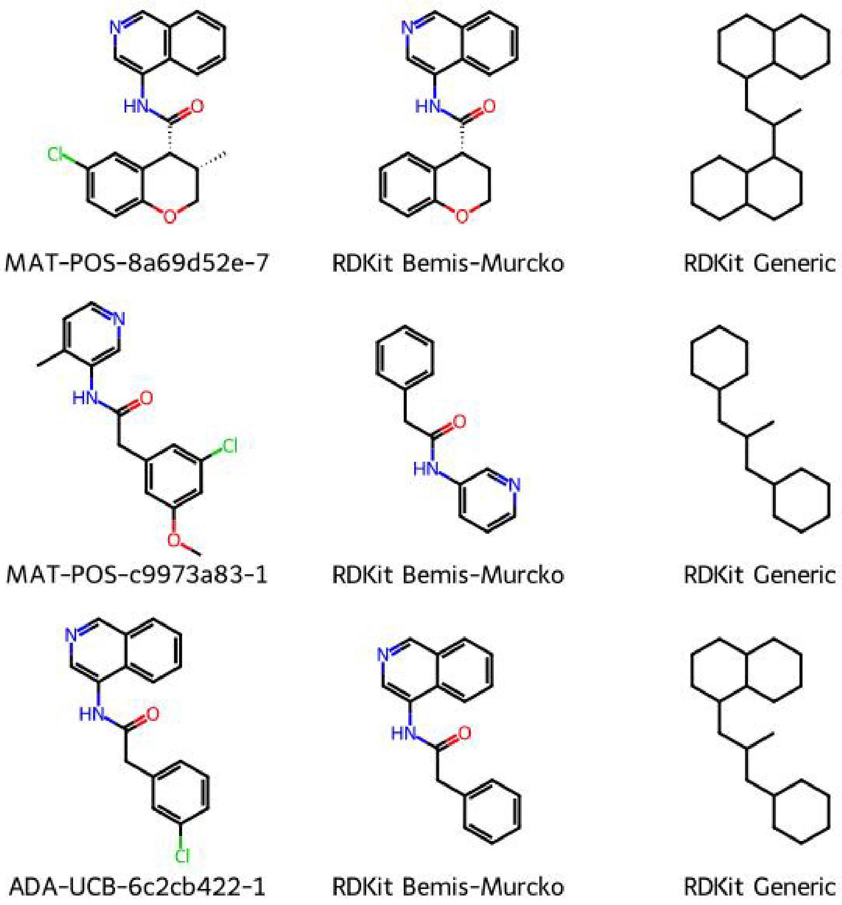

Figure S1: **Many different methods exist to automatically generate scaffolds from ligands.** The Bemis-Murcko scaffold implementations retain more information, but are sensitive to common scaffold modifications like nitrogen-walks. The generic scaffold still retains a chemically intuitive meaning of scaffold while grouping more molecules together.

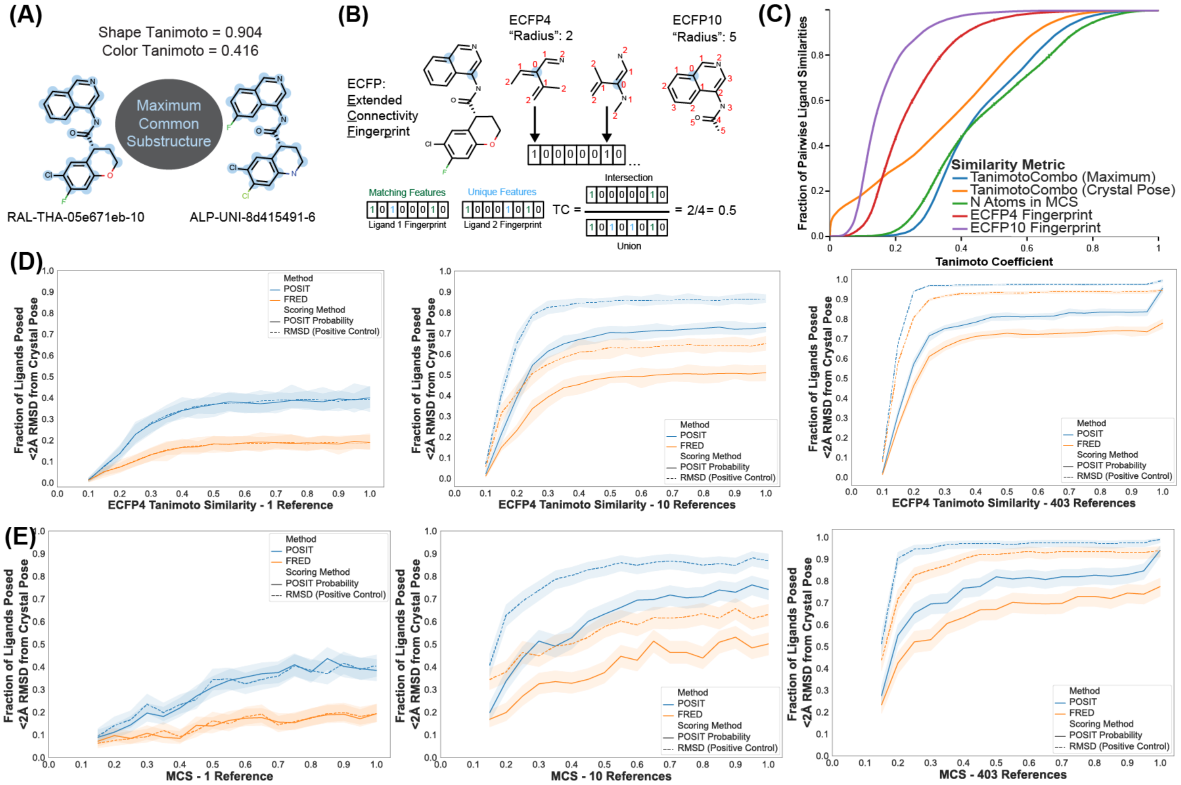

Figure S2: **Different common measurements of ligand similarity have differential impacts on analysis results.** (A) Maximum common substructure (MCS) and (B) Extended Connectivity Fingerprint (ECFP) are other common ways to compare ligand similarity. The Shape and Color Tanimoto scores for two example ligands from the COVID Moonshot are also shown for comparison. (C) The empirical cumulative distribution function plot of all pairwise Tanimoto Coefficients for each of the ligand similarity metrics shows how the choice of similarity metric can change the perception of how chemically diverse a ligand set is. The higher resolution ECFP10 suggests that the COVID Moonshot ligands are quite diverse, with >90% of the pairwise similarities <0.3, whereas the MCS and TanimotoCombo scores suggest that many of the ligands are quite similar. (D) The ECFP4 Tanimoto Similarity does not provide a useful description of this dataset, with most pairwise similarities between 0.1 and 0.4, causing the success rate to quickly plateau. (E) Although the MCS Tanimoto has a similar distribution to the TanimotoCombo, it also plateaus after about 0.5.

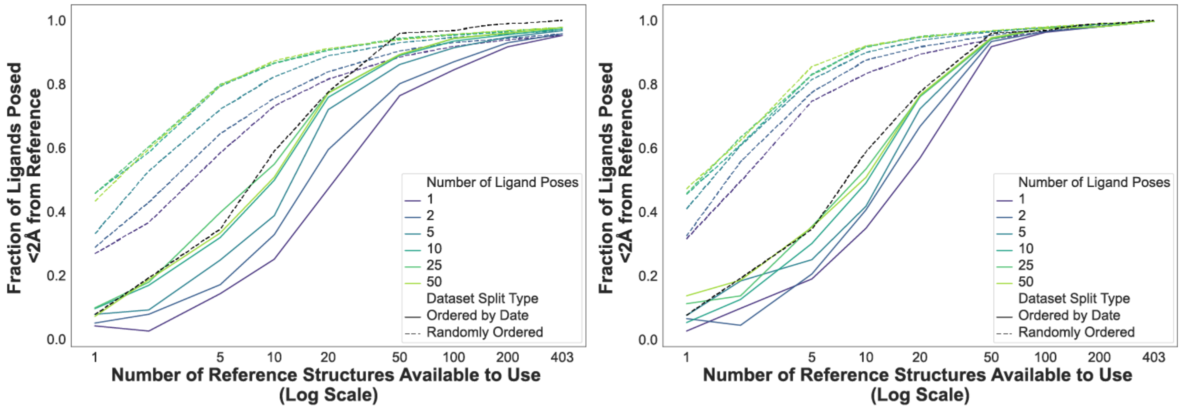

Figure S3: **Improved POSIT settings increase success rate comparable to RMSD ranking of the 50 poses.** The single pose results presented for most of the paper were run with the flags '–allow-retries' and '–relax-mode clash'. For the multipose analysis, these flags were removed in order to save time and compute. The figures are the same from Figure **??** with the addition of a black dotted line representing the Date Split, RMSD-scored POSIT results returning a single pose.

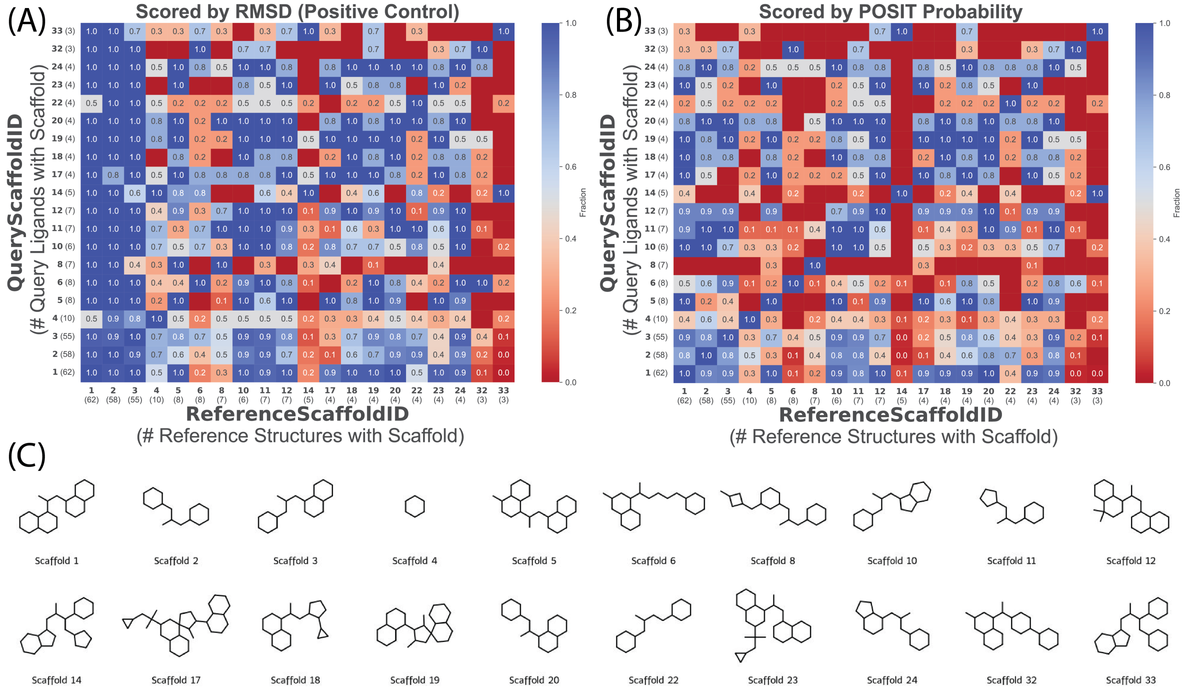

Figure S4: **Scaffold to scaffold cross-docking results analysis.** Heatmap of the fraction of ligands posed within 2Å of their crystal pose for the top 20 most represented scaffolds cross-docked (A) and (B). Each value represents the success rate of the query scaffold (each row) docked to the reference scaffold (each column). On the left (A) the poses are ranked by RMSD, and on the right (B) the poses are ranked by the POSIT Probability. C) The top 20 most represented scaffolds are generic Bemis-Murcko scaffolds are shown below.

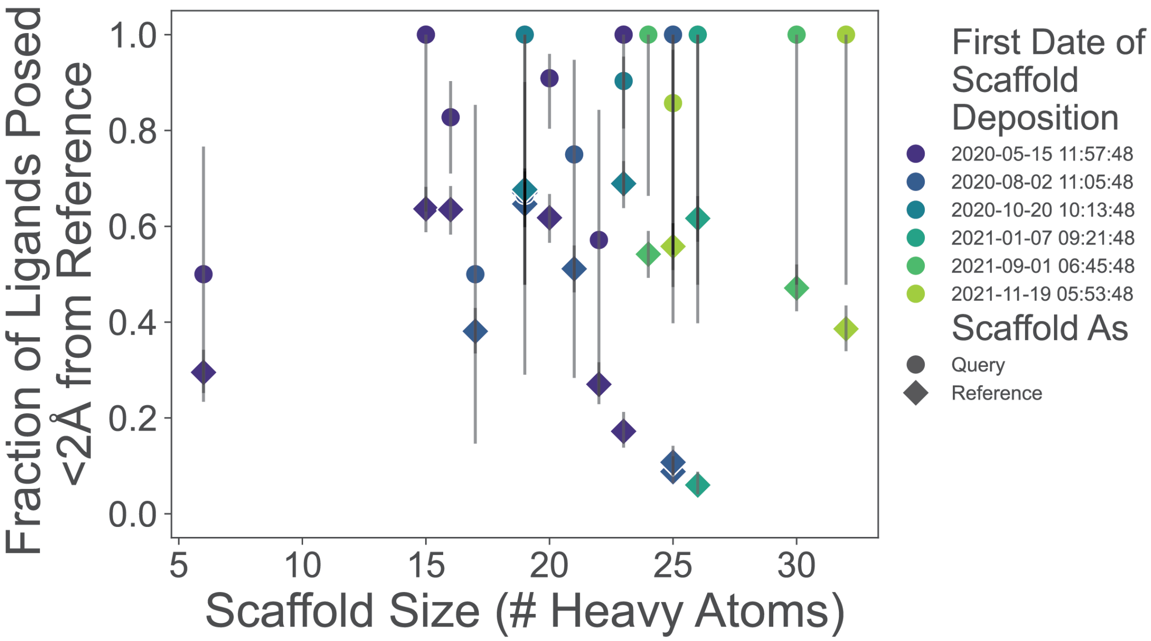

Figure S5: **Ligands were easier to pose than to use as reference.** Results from SI Figure S4 for the top 20 most represented scaffolds averaged for each scaffold, split by either using the scaffolds as query (circle) or reference (diamond). The data points are colored by the date on which the first structure for that scaffold was collected and error bars from bootstrapping over the available references are shown. In all cases, the performance of using a scaffold as the query performed better than using it as a reference.

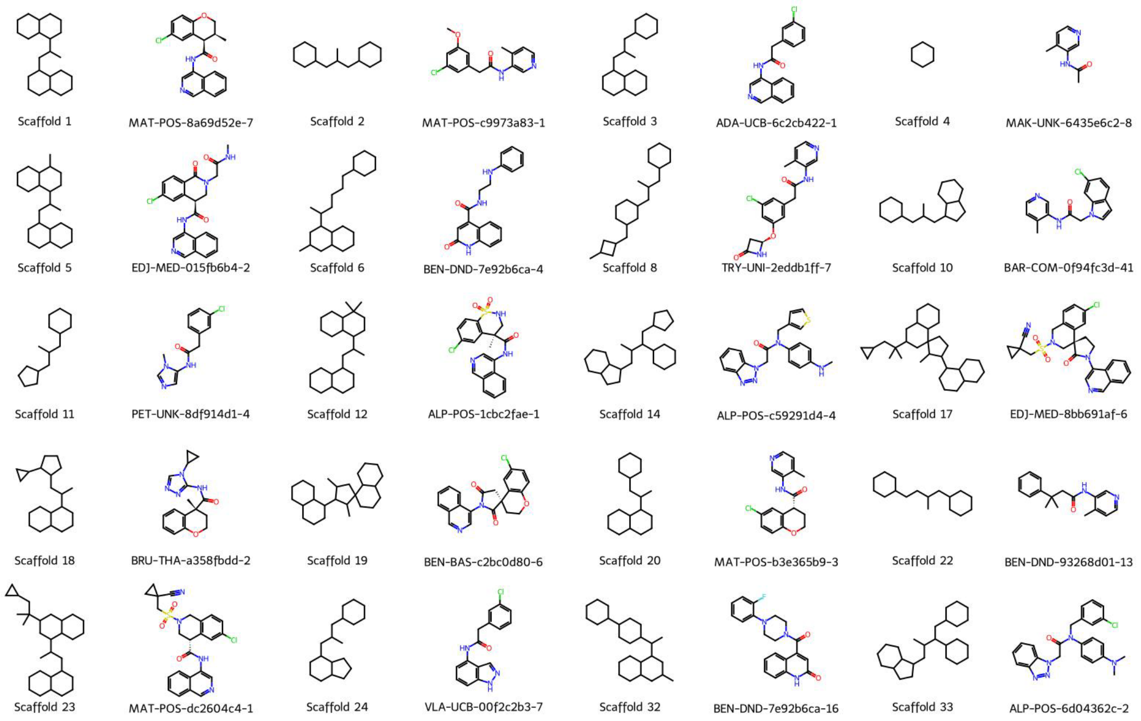

Figure S6: **Top 20 generic Bemis-Murcko scaffolds with examples of ligands from the COVID Moonshot.** The top 20 most represented scaffolds (left) used for SI Figure (S4-**??**) with representative examples of these scaffolds (right) pulled from the COVID Moonshot dataset. The generic Bemis-Murcko scaffold captures the core graph of the molecule while remove the heteroatom and bonding information. Several scaffolds are variations of one another—Scaffold 1 is only slightly modified in Scaffolds 5, 12, and 23.

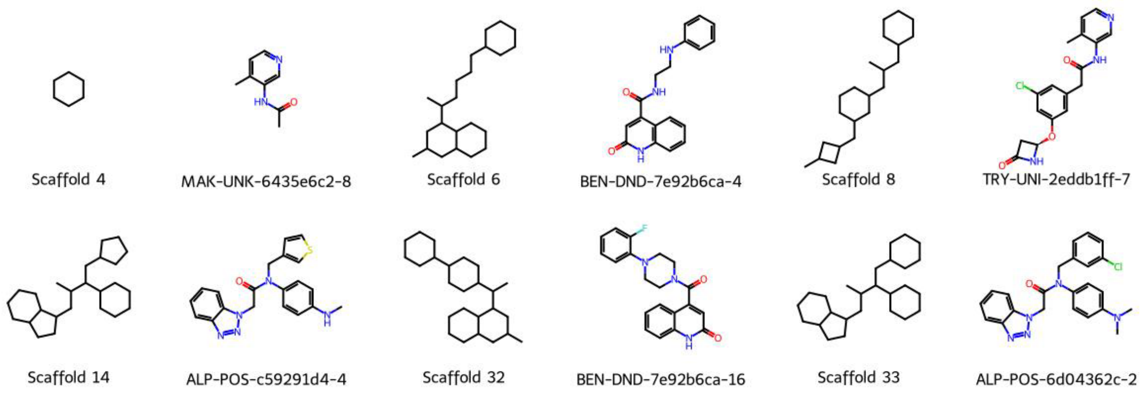

Figure S7: **Challenging scaffolds for pose prediction with examples of ligands from the COVID Moonshot.** The six worst-performing scaffolds (left) from the top 20 are shown alongside representative examples from the COVID Moonshot dataset (right). The fragment scaffold (Scaffold 4) was challenging both to dock and to use as a reference throughout this analysis.

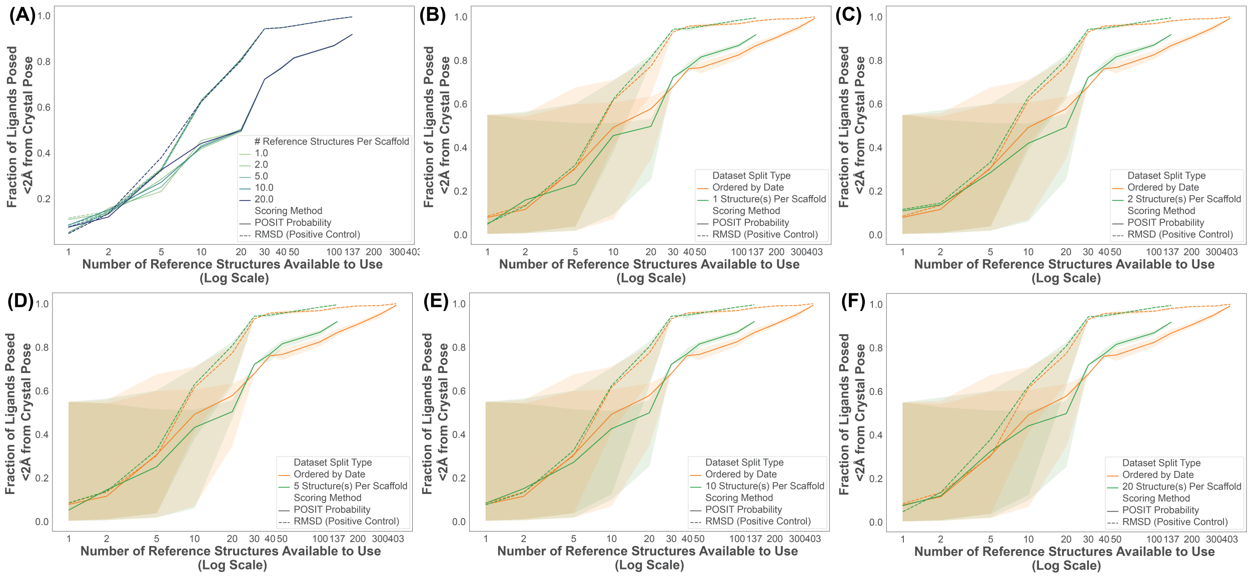

Figure S8: **Increasing the number of structures we collect per scaffold does not show any significant improvement in pose-prediction performance.** (A) The pose prediction performance, reported as the fraction of ligands posed ≤ 2 Åfrom the crystal pose, is plotted as a function of increasing the number of reference structures available to use, from 1 structure (light green) to 20 (dark blue) for either ranking by the POSIT Probability (solid) or RMSD (dashed). The results 1 structure per scaffold (B) to 20 structures per scaffold (F) in A are compared to the Temporal Split (orange).
